# Non-coding RNAs involved in the molecular pathology of Alzheimer’s disease: a systematic review

**DOI:** 10.3389/fnins.2024.1421675

**Published:** 2024-06-28

**Authors:** Reynand Jay Canoy, Jenica Clarisse Sy, Christian Deo Deguit, Caitlin Bridgette Castro, Lyoneil James Dimaapi, Beatrice Gabrielle Panlaqui, Wenzel Perian, Justine Yu, John Mark Velasco, Jesus Emmanuel Sevilleja, Anna Gibson

**Affiliations:** ^1^SciLore LLC, Kingsbury, TX, United States; ^2^Instiute of Biology, College of Science, University of the Philippines Diliman, Quezon City, Philippines; ^3^Center for Research and Innovation, Ateneo de Manila University School of Medicine and Public Health, Pasig City, Philippines; ^4^Cancer Immunology Program, Peter MacCallum Cancer Centre, Melbourne, VIC, Australia; ^5^Sir Peter MacCallum Department of Oncology, The University of Melbourne, Melbourne, VIC, Australia; ^6^National Institute of Molecular Biology and Biotechnology, College of Science, University of the Philippines Diliman, Quezon City, Philippines; ^7^Institute for Dementia Care Asia, Quezon City, Philippines; ^8^Institute of Molecular Biology and Biotechnology, National Institutes of Health, University of the Philippines Manila, Manila, Philippines; ^9^Mental Health Research Unit, National Center for Mental Health, Mandaluyong City, Philippines

**Keywords:** ncRNA, miRNA, lncRNA, circRNA, piRNA, Alzheimer’s disease, molecular pathology, biomarkers

## Abstract

Alzheimer’s disease (AD) is the leading cause of dementia globally, having a pathophysiology that is complex and multifactorial. Recent findings highlight the significant role of non-coding RNAs (ncRNAs), specifically microRNAs (miRNAs), long non-coding RNAs (lncRNAs), circular RNAs (circRNAs), and piwi-interacting RNAs (piRNAs) in the molecular mechanisms underlying AD. These ncRNAs are involved in critical biological processes such as cell proliferation, apoptosis, oxidative stress, amyloid-beta aggregation, tau phosphorylation, neuroinflammation, and autophagy, which are pivotal in AD development and progression. This systematic review aims to consolidate current scientific knowledge on the role of ncRNAs in AD, making it the first to encompass the four types of ncRNAs associated with the disease. Our comprehensive search and analysis reveal that ncRNAs not only play crucial roles in the pathogenesis of AD but also hold potential as biomarkers for its early detection and as novel therapeutic targets. Specifically, the findings underscore the significance of miRNAs in regulating genes involved in key AD pathways such as activin receptor signaling pathway, actomyosin contractile ring organization, and advanced glycation endproducts-receptor advanced glycation endproducts (AGE-RAGE) signaling pathway. This review also highlights the potential of ncRNAs in unveiling novel diagnostic and therapeutic strategies, emphasizing the need for further research to validate their clinical utility. Our systematic exploration provides a foundation for future bioinformatic analyses and the development of ncRNA-based precision medicine approaches for AD, offering new insights into the disease’s molecular pathology and paving the way for innovative treatment strategies.

**Systematic review registration:**

PROSPERO, https://www.crd.york.ac.uk/prospero/, CRD42022355307.

## Introduction

Alzheimer’s disease (AD) is the most prevalent form of dementia, accounting for 60%–80% of cases worldwide (Scheltens et al., 2016; Alzheimer’s Association, 2023). In 2019, the global cost of dementia reached $1.3 trillion, with approximately 50% attributed to informal care (Wimo et al., 2023). According to the estimate of World Health Organization (2023), over 55 million people are living with dementia, with an additional 10 million new cases added each year. Notably, more than half of these cases (60%) originate from low- and middle-income countries, highlighting the substantial economic burden that dementia imposes. From 1990 to 2019, the incidence of disability adjusted life-years (DALYs) due to AD and other forms of dementia increased from 9.7 to 25.3 million worldwide (Li X. et al., 2022).

Dementia is a general term that refers to the decline in cognitive function severe enough to interfere with daily life and activities (National Institute on Aging, 2022). This decline predominantly affects thinking, memory, and reasoning functions—leading to a wide array of symptoms with varying degrees of severity. These symptoms include memory loss, difficulty in communication or finding words, challenges with visual and spatial abilities, trouble solving problems or reasoning, inability to handle complex tasks, difficulties in planning and organizing, confusion, disorientation, and changes in personality leading to agitation or withdrawal (National Institute on Aging, 2022; Alzheimer’s Disease and Dementia, 2023). While AD accounts for most cases of dementia, other types also exist including frontotemporal dementia, Lewy body dementia, vascular dementia, and mixed dementia, which can be a combination of two or more types.

Previously, definitive diagnosis for AD was only through the postmortem detection of amyloid-beta plaques and neurofibrillary tangles in autopsied brain tissues (McKhann et al., 1984; Scheltens et al., 2016). However, the research framework provided in 2018 by the National Institute on Aging and the Alzheimer’s Association (NIA-AA Research Framework) broadened the definition and diagnostic criteria of AD by introducing the use of *in vivo* biomarkers (Jack et al., 2018). These *in vivo* biomarkers can be imaging and biofluids that measure amyloid-beta deposition, pathologic tau, and neurodegeneration (ATN framework). Indeed, this biological definition of AD has allowed the definitive diagnosis in living patients, enabling the development of scientific research geared toward a comprehensive understanding of the disease.

Based on the age of onset, AD can be categorized as early-onset (EOAD) if the disease develops before the age of 65, or as late-onset (LOAD) if it manifests at 65 years old or later. Among AD cases, EOAD accounts for only 5%–6% while the rest are LOAD (Mendez, 2019). In terms of clinical characteristics, EOAD and LOAD are similar except in baseline cognition, rate of cognitive decline, and survival duration (Seath et al., 2023). In terms of genetic etiology, EOAD is clearly defined while LOAD is less definitive (Long and Holtzman, 2019; Uddin et al., 2021; Andrade-Guerrero et al., 2023). EOAD is linked to the autosomal dominant mutations in *amyloid-beta precursor protein* (*APP*), *presenilin-1* (*PSEN1*), and *presenilin-2* (*PSEN2*). In contrast, while many genes are associated with the different aspects of LOAD, they do not provide a comprehensive explanation for its etiology. As such, there is still this huge need to fully elucidate the origins of LOAD. Having a deeper comprehension of its etiology might pave the way for its early detection, novel therapeutic interventions, and better disease management.

There are a few systematic reviews and meta-analyses that have evaluated the association of different lifestyle factors with AD pathophysiology. These factors include physical activity or the lack thereof (Zhu et al., 2015; Stephen et al., 2017; Demurtas et al., 2020; Andrade et al., 2022; López-Ortiz et al., 2023), the use of non-steroidal drugs (Etminan et al., 2003), and diet (Muñoz Fernández et al., 2017; Yusufov et al., 2017; Xu Lou et al., 2023). Other systematic reviews involve the plasma nutrient status of patients with AD (Lopes da Silva et al., 2014), the prevalence of the disease and neuropsychiatric symptoms (Zhao Q. F. et al., 2016; Niu et al., 2017), neuroimaging markers (Bloudek et al., 2011; Ferreira et al., 2011; Liu et al., 2015), potential pharmacological treatment of the disease (Kaduszkiewicz et al., 2005; Hyde et al., 2013; Lu et al., 2020; David et al., 2022), and infection (Ou et al., 2020).

The limited understanding of the etiology of the most prevalent type of AD might be attributed to the focus on protein-coding genes in previous disease studies. However, over the past decade, the vital role of noncoding RNAs (ncRNAs) in AD’s pathophysiology has started to emerge (Maoz et al., 2017; Jain et al., 2019; Shobeiri et al., 2023). ncRNA are functional RNA molecules that are not translated into proteins. They are broadly categorized into housekeeping ncRNAs and regulatory ncRNAs. Housekeeping ncRNAs are essential for basic cellular functions, while regulatory ncRNAs are crucial in gene expression which affects various cellular activities. Major classes of ncRNA include, microRNAs (miRNAs), long noncoding RNAs (lncRNAs), circular RNAs (circRNAs), and piwi-interacting RNAs (piRNAs) (Liu et al., 2023). These ncRNAs were found to play key roles in development, immunity, cell proliferation, apoptosis, oxidative stress, amyloid-beta aggregation, tau phosphorylation, neuroinflammation, and autophagy, which all contribute to AD development (Chen et al., 2019; Zhang et al., 2021). They constitute a class of RNA molecules that, despite not encoding proteins, interact with DNA, RNA, proteins, and even other ncRNAs to modulate a wide range of biological processes, such as gene transcription, RNA turnover, mRNA translation, and protein assembly.

Specifically, miRNAs, lncRNAs, circRNAs, and piRNAs have attracted significant attention for their interaction with diverse molecular entities and for their pivotal roles in the regulation of gene expression (Nikolac Perkovic et al., 2021). In particular, miRNAs were found to influence the expression of genes involved in amyloid-beta deposition, tau phosphorylation, and neuroinflammation (Lei et al., 2015; Zhong et al., 2018). miRNAs are formed when miRNA genes are transcribed by RNA polymerase II to form long primary miRNA (pri-miRNA) transcripts. These pri-miRNAs undergo processing through DROSHA and DICER to form a miRNA duplex, which is loaded into Argonaute proteins that forms mature miRNAs (Komatsu et al., 2023). Likewise, lncRNAs are also transcribed by RNA polymerase II from intergenic, exonic, or distal protein-coding regions of the genome. These transcripts are capped with a 5′ methyl-cytosine cap and a 3′ poly (A) tail to form lncRNAs (Dhanoa et al., 2018; Loganathan and Doss, 2023). LncRNAs were observed to regulate amyloid-beta production, tau phosphorylation, and synaptic function in AD (Zhang et al., 2021). Interestingly, they were also found to bind to miRNAs or proteins and can therefore modulate their activity. In the same sense, circRNAs were observed to function as miRNA sponges that affect AD onset and progression (Zhao Y. et al., 2016; Kristensen et al., 2019). They are formed through the backsplicing of canonical splice sites. Specifically, exons are spliced in the opposite direction and combines the upstream and downstream 3′ and 5′ splice sites, which forms circRNA (Nisar et al., 2021; Loganathan and Doss, 2023). Numerous piRNAs have also been found to be differentially expressed in AD brains. Most of these were linked with genome-wide significant AD risk single nucleotide polymorphisms (SNPs) in the *apolipoprotein E* (*APOE*) cluster. piRNAs were also linked with the dysregulation of different AD-associated pathways, such as *pituitary adenylate cyclase activating polypeptide* (*PACAP*) signaling in neurons, axonal transport, and *α-amino-3-hydroxy-5-methyl-4-isoxazolepropionate* (*AMPA*) receptor trafficking (Sato et al., 2023). RNA precursors transcribed in the nucleus are exported into the cytoplasm to form piRNA precursors which undergo primary and secondary amplification cycles to form mature piRNAs that form complexes with PIWI proteins (Loganathan and Doss, 2023).

In this paper, our overall objective is to systematically review all scientific studies that evaluate the role of ncRNAs in the brain and circulating tissues of individuals with confirmed AD. To the best of our knowledge, this is the first systematic review which consolidates the four types of ncRNAs (miRNAs, lncRNAs, circRNAs, and piRNAs) that are associated with AD. This systematic review further looks at some of the top significant miRNAs associated with AD, among the associated ncRNAs, and to discover how it contributes to AD pathology. In addition, the consolidated lists of lncRNAs, circRNAs, and piRNAs hold potential for future bioinformatic analyses to further elucidate the role of ncRNAs as biomarkers and therapeutic targets of AD. Understanding the roles of ncRNA in AD may be pivotal in developing treatment strategies that prevent the progression of the disease through precision medicine.

## Materials and methods

**Figure 1:**
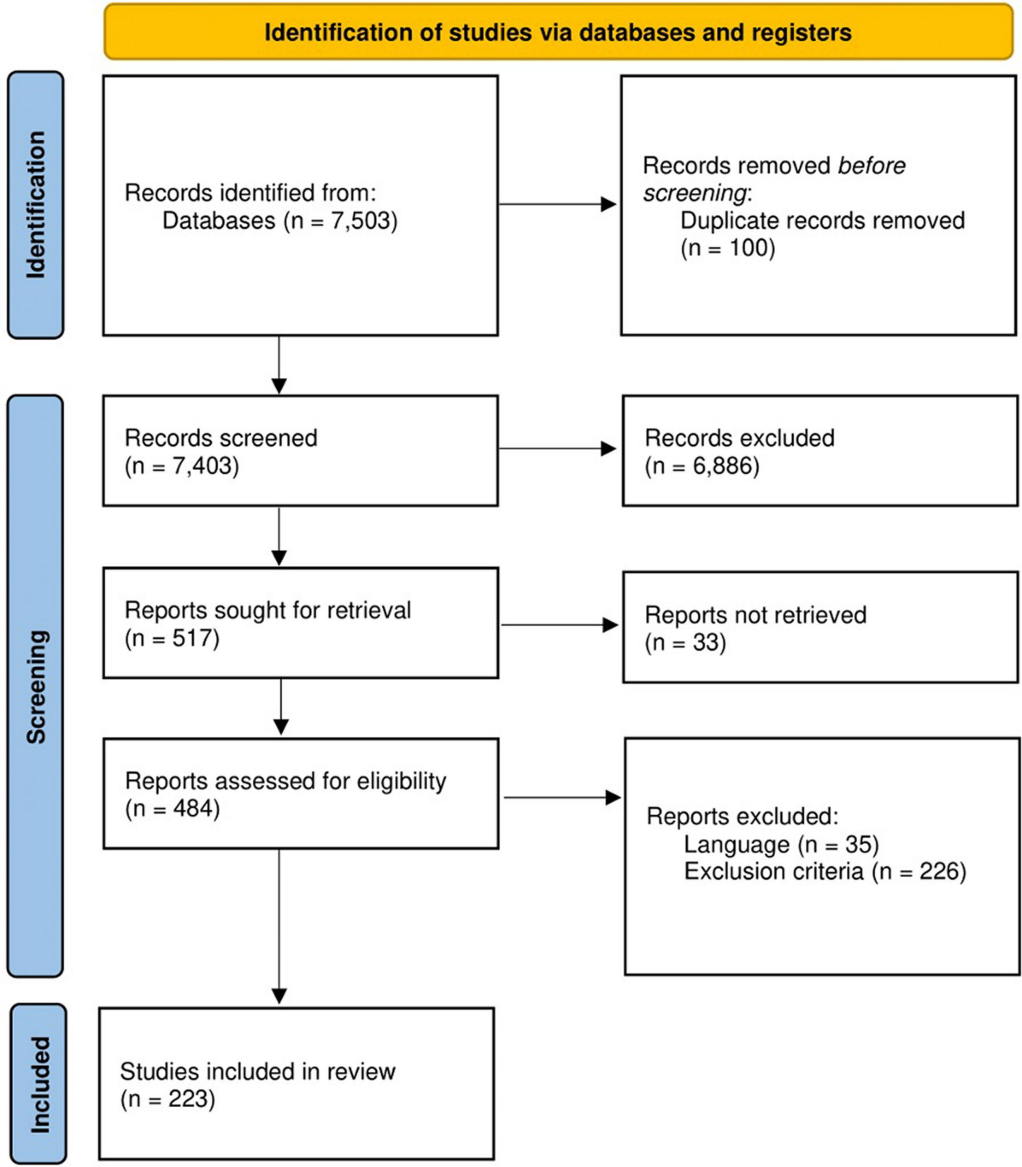
Workflow utilized in this systematic review as adopted from PRISMA. At the start, 7,503 studies were retrieved from PubMed, Google Scholar, and Scopus. After the title and abstract screenings, the retrieved studies were reduced to 484 and were subjected further for full-text screening following set inclusion and exclusion criteria. Finally, a total of 223 studies were included in this systematic review for downstream analyses.

This systematic review implemented a workflow (Figure 1) following the Preferred Reporting Items for Systematic Reviews and Meta-Analyses (PRISMA) (Page et al., 2021). The protocol was registered to PROSPERO with registration number: CRD42022355307.

### Search strategy

Database searches were conducted on PubMed, Google Scholar, and Scopus, which are commonly accessed databases that contain peer-reviewed biomedical literature, in 21–23 July 2023 by three independent reviewers. Different keywords were used to search each database which are provided in Supplementary Table 1. All results of each database search, regardless of publication year and study design, were exported and compiled by a fourth independent reviewer. Duplicates of studies were removed before proceeding to abstract screening.

### Abstract screening

The abstracts of each article from the compiled list were used as basis for further inclusion or exclusion from the study. Specifically, the studies included for further screening were those that were published in English and were independent original research that evaluated the role of ncRNAs on the development of AD. The studies that were excluded were reviews, case reports, abstracts, letters, commentaries, methodological studies, conference poster presentation, or any article that was not peer-reviewed. Those that did not involve human research subjects or were not accessible were also excluded. The liberal acceleration policy was used, such that excluded studies were screened by a second independent reviewer. Any disagreement was settled by a third independent reviewer.

### Full text screening and data extraction

The included studies were further assessed, and the data for each study was extracted by an independent reviewer. Apart from the criteria presented in the abstract screening, studies that did not specify the type of dementia in their study population were excluded. Studies were also excluded if the type of ncRNA was not specified, and if there were no control group present in the study.

Google Forms was used to record the following information: basic study information (authors, title, year of publication, DOI, and journal published), participant demographics (total number of cases and controls, mean age, number of males and females, race), tissue samples, and methodology/technology used. Data related to ncRNA expression profiles was also extracted, including ncRNA gene expression fold change, *p*-values, and the functions of the ncRNAs, when available. The liberal acceleration policy was also implemented for full-text screening. Any discrepancies were adjudicated by a third independent reviewer.

### Quality appraisal

Each study after data extraction was appraised of its quality by two independent researchers using the Quality of Genetic Association Studies Tool (Q-Genie) tool (Sohani et al., 2015). This tool was specifically developed to identify high quality genetic studies, and it has been shown to exhibit good inter-rater reliability, internal consistency, and overall reliability. It consists of 11 questions which are answered using a Likert scale ranging from 1 (poor) to 7 (excellent). The questions were classified into nine categories: rationale for the study, sample selection, exposure classification, outcome classification, identification of sources of bias, presentation of the statistical plan, quality of statistical methods, testing of assumptions in genetic studies, and result interpretation. The studies were scored and categorized as low, moderate, and high-quality studies based on their average total scores. Those studies with scores ≤ 35 were considered low-quality studies, >35 to ≤45 were of moderate quality, and > 45 were high quality studies. The low-quality studies were then subsequently excluded from this systematic review. All included studies (223) were compiled in Supplementary Table 2.

### miRNA gene-target prediction and pathway enrichment analysis

Potential gene targets of the miRNAs obtained from the literature review were identified using the multiMiR package (version 3.18) (Ru et al., 2014). The multiMiR package facilitates the identification of gene targets of miRNAs from 14 validated and predicted miRNA-target interaction databases. As required by the package, only miRNAs with precursor-derived arm designation information (i.e., “-3p” or “-5p” suffix) were included in the analysis. miRNA-gene target pairs with an “all.sum score” of at least 5 (indicating presence in at least 5 of the databases queried), were considered significant.

Of the significant miRNAs, only the top 3 with the highest number of gene targets were subjected to exploratory functional analysis via the STRING 12.0 database (Search Tool for the Retrieval of Interacting Genes/Proteins; https://string-db.org) (Szklarczyk et al., 2023). Specifically, the target genes of the top 3 miRNAs were subjected to pathway enrichment analysis to elucidate the potential molecular pathways associated with these miRNA gene targets. Only the enriched pathways from Gene Ontology (Biological Processes), KEGG and Reactome classification systems were recorded. Protein–protein interaction network analysis was also performed to visualize and appraise the biological connectivity of the gene targets.

## Results

This systematic review shows that the research on ncRNAs in the context of AD has grown significantly over the years (Supplementary Figure 1). All studies included in this systematic review involved the detection or validation of ncRNAs in individuals with AD, compared with neurotypical controls. The sample sizes of these included studies ranged from one to 1,021 cases and from one to 1,385 control participants and their ages ranged from 58 to 93 years (Supplementary Figure 2).

**Figure 2:**
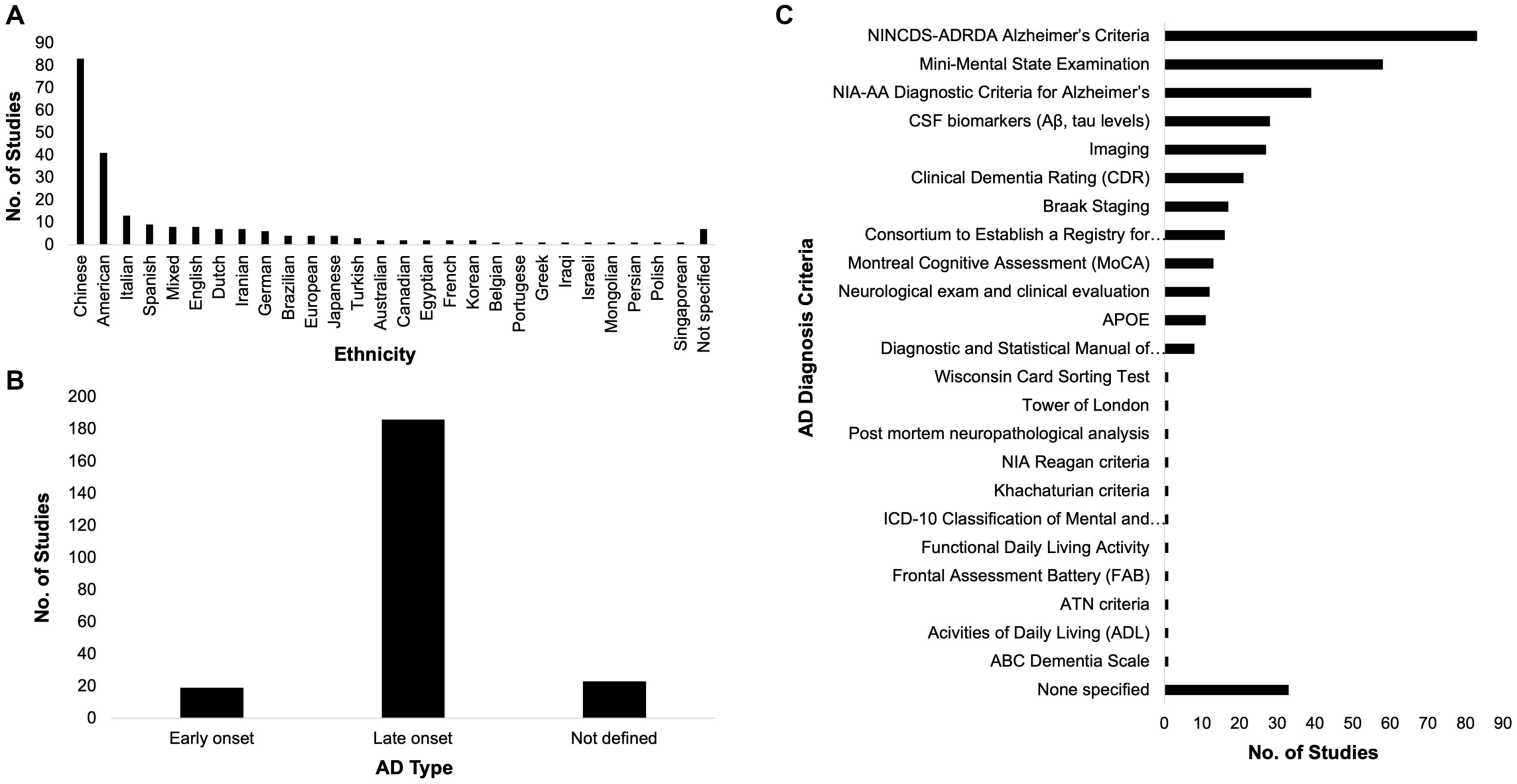
Race, Alzheimer’s disease (AD) type, and diagnosis criteria. The participants across the included studies in this systematic review span different ethnicities **(A)**. Most of the included studies dealt with the late-onset type of AD **(B)** and most of them utilized at least two AD diagnosis criteria **(C)**.

The race of participants across these studies were varied, although dominated by East Asians, Europeans, and Americans (Figure 2A). Among the included studies, 19 categorically classified their participants or samples as EOAD while 186 studies classified theirs as LOAD (Figure 2B). At least two AD diagnosis criteria were used by most of the studies (Figure 2C). Popular among AD diagnosis criteria utilized were the National Institute of Neurological and Communicative Disorders and Stroke and the Alzheimer’s Disease and Related Disorders Association (NINCDS-ADRDA) Alzheimer’s Criteria, the Mini Mental State Exam (MMSE), and the NIA-AA Diagnostic Criteria for Alzheimer’s.

**Figure 3:**
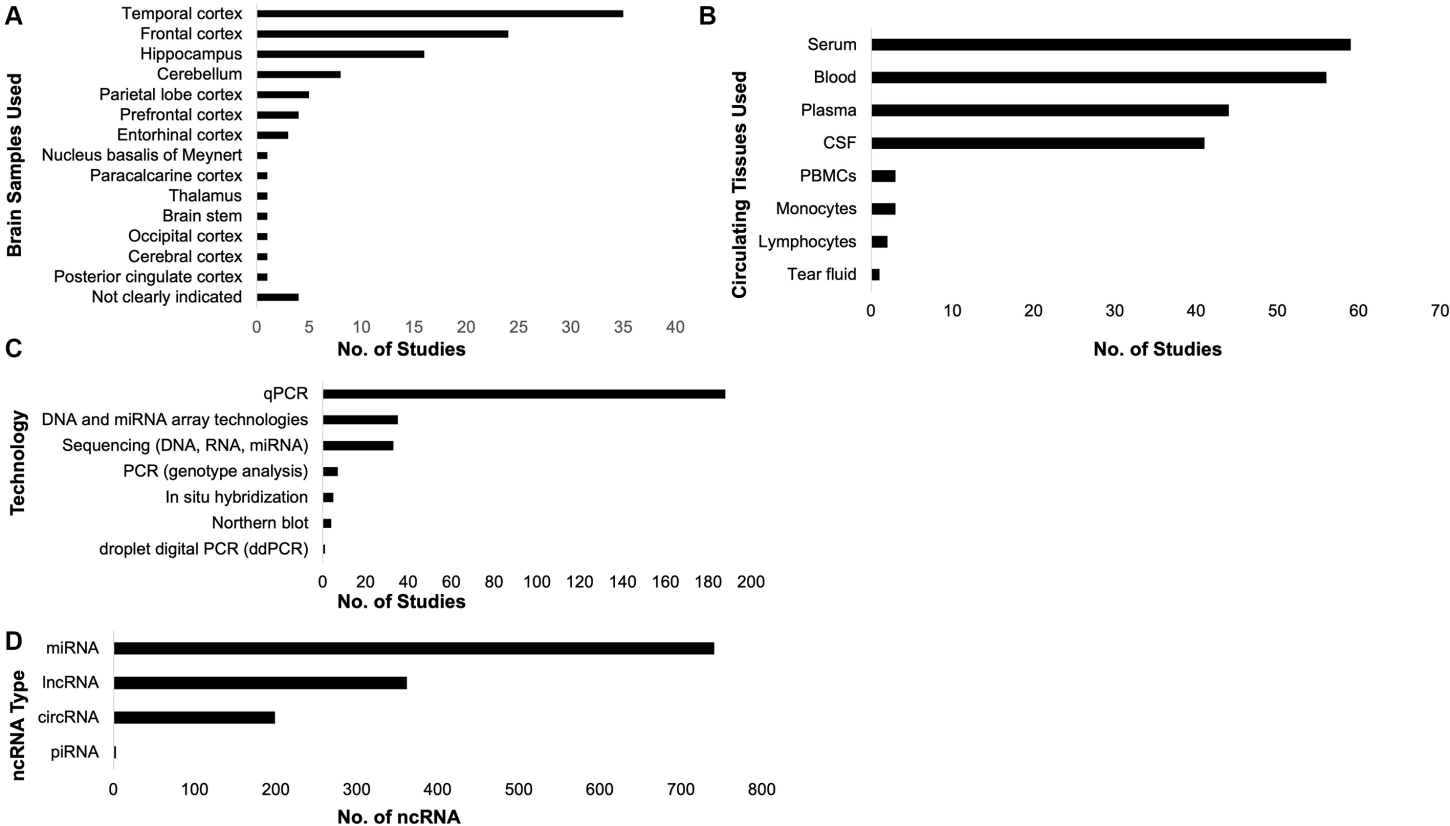
Samples collected, technologies utilized, and non-coding RNAs (ncRNAs) compiled. The included studies in this systematic review collected brain **(A)** and circulating **(B)** tissues. Most of the studies utilized qPCR to investigate the role of ncRNAs in Alzheimer’s disease **(C)**. From the included studies, the unique ncRNAs associated with AD pathology were compiled **(D)**.

To investigate the ncRNAs with respect to AD, brain and circulating tissue samples (Figures 3A,B) were collected by the included studies, where most of them used the qPCR technology (Figure 3C) to investigate the role of ncRNAs in AD pathology. From these, unique miRNAs, lncRNAs, circRNAs, and piRNAs were compiled (Figure 3D; Table 1; Supplementary Table 3). It is interesting to note that some of these studies did not just investigate only one ncRNA type. There was one study that analyzed lncRNAs and circRNAs, three studies that analyzed miRNAs and circRNAs, three studies that studied miRNAs and lncRNA, and one study that studied miRNAs and piRNAs together. These studies have also suggested that the each ncRNA type affects AD pathophysiology through different mechanisms, such as amyloid-beta accumulation, tau phosphorylation, cell apoptosis, and neurotoxicity (Table 1).

**Table 1:** Summary of unique ncRNAs included in the review.

ncRNA type	No. of unique ncRNA	Common described functions
miRNA	741	Affects CSF tau and amyloid-beta levels (Guévremont et al., 2022); modulates *BACE1* expression (Zeng et al., 2019; Dakterzada et al., 2020); involvement in the MAPK signal pathway (Lu et al., 2021); binds to several associated key genes, such as *APP, NGF, USP25, PDRG1, SMAD4, UBQLN1, SMAD2, TP73, VAMP2, HSPBAP1,* and *NRXN1* (Kumar and Reddy, 2018); regulates cell apoptosis and GTDC-1/CDK-5/Tau phosphorylation signaling mechanism (Liu and Zhang, 2019); regulates *APOE4*, secretases and *APP* expression; increases neuroinflammation (Idda et al., 2018; Liu S. et al., 2022)
lncRNA	362	Produces amyloid-beta plaques and *APP* (Feng et al., 2018; Asadi et al., 2021; Greco et al., 2022); promotes apoptosis and oxidative stress (Ding et al., 2022); increases tau phosphorylation (Guan et al., 2022); increases the levels of mRNA involved in glial-derived neurotrophic factor (GDNF) and ephrin receptor B2 (EPHB2) which are implicated in AD pathogenesis (Idda et al., 2018)
circRNA	199	Mediates amyloid-beta induced neurotoxicity (Pan et al., 2022; Chen and Fang, 2023); promotes amyloid-beta-25-35 induced apoptosis, inflammation, and oxidative stress (Li et al., 2020; Wu et al., 2021); affects synaptic plasticity and memory retention (Song et al., 2022)
piRNA	3	Affects cellular homeostasis during oxidative stress (Qiu et al., 2017; Mao et al., 2019); contributes to the exacerbation of cognitive deficit and inhibits clearance of amyloid-beta-42

To illustrate the potential of our compiled ncRNA list, we focused on the 150 miRNAs that were found to be highly significant (*p* < 0.001) by their respective studies. We performed gene target prediction analysis on all the miRNAs and identified the top 3 miRNAs (*let-7b-5p*, *let-7 g-5p*, and *mir-15b-5p*) with the most gene targets. Pathway enrichment analysis (Supplementary Table 4) of the gene targets of the top miRNAs uncovered pathways that can be related to AD pathology such as the Activin Receptor Signaling Pathway concerning the protection and survival of neurons, the Actomyosin Contractile Ring Organization which can be linked to neurons re-entering the cell cycle that leads to apoptosis, and the advanced glycation end products-receptor advanced glycation endproducts (AGE-RAGE) Signaling Pathway.

## Discussion

The increasing number of published studies over the years have shown the great potential of ncRNAs as biomarkers and therapeutic targets of AD. Literature reviews that highlight the role of ncRNAs in AD have also been published in recent years (Lukiw, 2007; Zhang et al., 2021; Yoon et al., 2022; Olufunmilayo and Holsinger, 2023; Pierouli et al., 2023). These studies hypothesize the role of ncRNAs in disease pathogenesis by examining the involvement of ncRNAs in amyloid-beta peptide and tau accumulation, neuroinflammation, synaptic dysfunction, neuronal apoptosis, and neuronal loss (Liu Y. et al., 2022; Li L. et al., 2023; Olufunmilayo and Holsinger, 2023). Other literature reviews also examined the potential of ncRNAs as biomarkers for diagnosis (Swarbrick et al., 2019; Noor Eddin et al., 2023; Shobeiri et al., 2023).

### miRNAs

Majority (57%) of the compiled ncRNAs in this systematic review are miRNAs, some of them have been implicated in the amyloid-beta pathway. Specifically, miRNAs affect *APP* and the enzymes that produce amyloid-beta. *MiR-16, miR-29a/b-1* and *c*, *miR-186*, and *miR-195* have been shown to be downregulated in AD patients and AD mice models (Hébert et al., 2009; Zhu et al., 2012; Lei et al., 2015; Kim et al., 2016; Zhong et al., 2018). *MiR-16* affects the expression of APP and *beta-secratase 1* (*BACE1*), which are enzymes involved in the production of amyloid-beta, and it has also been shown to decrease tau phosphorylation in neuronal cell lines (Parsi et al., 2015). *MiR-29a* and *29b-1* were decreased in AD subjects with increased *BACE1* (Hébert et al., 2009), and *miR-195* was also decreased in AD patients who were carriers of the epsilon 4 allele of *apolipoprotein E* (*ApoE ε4*; Cao et al., 2021). The *ApoE ε4* affects the phospholipid composition and neuronal homeostasis of the brain. It plays a role in amyloid-beta clearance, neurofibrillary tangle accumulation, and synaptic plasticity. miRNAs also play a role in neuroinflammation. *MiR-132* and *miR-212* were upregulated in AD patients and led to the decrease in *silent information regulator 1* (*SIRT1*) expression, which has anti-inflammatory roles in AD (Hadar et al., 2018).

As it is a main hallmark of AD, miRNAs have been investigated as novel biomarkers due to their diverse functions. The downregulation of miRNAs results in the upregulation of AD-related genes, such as *APP*, *PSEN1,* and *PSEN2*. Expression changes for miRNAs were found in AD patients’ brains and cerebrospinal fluid (CSF). Peripheral blood miRNAs were also being explored as biomarkers, but there is a need to further validate these studies. According to Wu et al. (2015), 20 distinct miRNAs were upregulated and 32 miRNAs were significantly downregulated in AD patients, compared to controls. The *miR-29b, miR-181c, miR-15b, miR-146a*, and *miR-107* were consistently found to be downregulated in at least two independent studies (Wu et al., 2015). *MiR-26b-5p, miR-615-3p, miR-4722-5p*, and *miR23a-3p* were also selected as miRNAs with the highest accuracy for the diagnosis of the AD group, compared to those with Parkinson’s disease (Zotarelli-Filho et al., 2023). In a meta-analysis involving 10 studies, the diagnostic accuracy of miRNAs yielded a combined area under the curve (AUC) of 0.88, with a pooled sensitivity of 0.80, and the specificity of discriminating cases from controls was 0.83 (Zhang et al., 2019). Similar results were obtained in a separate study by Hu et al. (2016) which included 7 seven studies; a pooled sensitivity of 0.86, pooled specificity of 0.87 and AUC of 0.87 was reported. These findings suggest that miRNAs are excellent biomarkers for the diagnosis of AD (Hu et al., 2016; Zhang et al., 2019).

### lncRNAs

The second most studied ncRNAs are lncRNAs. LncRNAs can impact AD pathogenesis through chromatin modulation, post-transcriptional and post-translational regulation, and protein complex organization (Luo and Chen, 2016; Zhang, 2016). As such, it is being explored as a biomarker for AD. Similar to miRNAs, lncRNA also affect the expression of *BACE1*. The *antisense transcript of BACE1* (*BACE1-AS*) is a lncRNA that upregulates *BACE1* and is highly expressed in patients (Modarresi et al., 2011; Zhang et al., 2018). Specifically, *BACE1-AS* affects *BACE1* stability through the formation of an RNA duplex that results in the upregulation of *BACE1* (Zeng et al., 2019). The cortex of patients with AD showed increased levels of *APP*, *BACE1*, *BACE1-AS*, and amyloid-beta (Kang et al., 2014). Due to its effect, *BACE1-AS* has also been explored in mice models. Knockdown models of *BACE1-AS* by siRNA resulted in reduced *BACE1*, *APP* production, and tau phosphorylation in the hippocampus in SAMP8 mice, a neurodegeneration model used to study AD (Zhang et al., 2018). Overall, this led to enhanced memory and improved learning behavior. The lncRNA *X inactive specific transcript* (*XIST*) has also been found to regulate the expression of BACE1, and was found to be increased in AD mice and cell models, compared to controls (Yue et al., 2020; Yan et al., 2022). *XIST* knockdowns in mice hippocampal neurons showed reduced amyloid-beta-induced neurotoxicity, neuroinflammation, apoptosis, and oxidative stress. lncRNAs in plasma are also being explored as biomarkers. Differential expression of *AL133415.1, AC020916.1,* and *ASMTL-AS* were significantly lower in AD patients compared to controls in the Chinese population (Cheng et al., 2023). LncRNAs have also been explored as therapeutic targets for AD. The *nuclear enriched abundant transcript 1* (*NEAT1*) is a lncRNA that plays a role in neuroplasticity and long-term memory formation (Asadi et al., 2021). In APP/PS1 transgenic mice, there has been an increased level of *NEAT1*, which caused amyloid-beta accumulation and impaired cognitive skills (Huang et al., 2020). It served as a sponge for *miR-107*, causing a lower expression of *miR-107* which leads to tau phosphorylation *in vitro* (Ke et al., 2019). The knockdown of *NEAT1* mitigated the effects of the miRNA by upregulating *miR-107*. It was also found to target *miR-193a*, which reduced apoptosis, inflammatory cytokine levels, and oxidative stress in mice models (Li Y. et al., 2023). This suggests that *NEAT1* may serve as a suitable target for the treatment of AD.

### circRNAs

circRNAs serve as sponges of miRNAs and influence AD onset and progression. The circular RNA *ciRS-7* is one of the most characterized circRNA which contains binding sites for *miR-7* (Zhao Y. et al., 2016; Kristensen et al., 2019). A decrease in *ciRS-7* has been detected in AD brains which lead to a decrease in the sponging activity of *miR-7*. Overall, this results in an increase in *BACE1* and *APP* production and decrease in *ubiquitin protein ligase A* (*UBE2A*), a protein that hinders amyloid-beta production. A separate study has also identified *circAXL, circGPHN,* and *circPCCA* and their predictive value for AD through a receiver operating characteristic (ROC) curve and AUC analyses (Li et al., 2020). These circRNA were correlated with MMSE scores, amyloid-beta-42 levels, and t-tau levels. Their mechanism of action is hypothesized to affect the transcription of their parental genes, which are critical in neuroinflammation. They are hypothesized to regulate neuroinflammation and neuronal cell death by competitively binding to miRNAs that also increase AD risk. *CircKCCN2, circHOMER2, circFMN1, circDOCK1, circMAP7, circRTN4,* and *circPICALM* were also correlated with Braak scores (Zhang and Bian, 2021). Overall this suggests that circRNA may serve as potential novel biomarkers and therapeutic targets for AD.

### piRNAs

piRNAs silence transposable elements in genes, and regulate gene expression by interacting with PIWI-clade Argonaute proteins (Olufunmilayo and Holsinger, 2023). In AD, more extensive research on the roles of piRNAs are necessary. However, there are a few studies which have identified the role of piRNA in AD pathophysiology. piRNAs promote oxidative stress and amyloid-beta and tau protein accumulation, which contribute to the onset and progression of AD. Specifically, *piR-34393* and *piR-38240* were found to decrease *cytochrome C somatic* (*CYCS*) and *karyopherin subunit alpha 6* (*KPNA6*) expression, which have both been correlated to AD (Roy et al., 2017). A total of 103 differentially expressed piRNAs have also been identified that were associated with SNPs in AD cases, compared to controls (Qiu et al., 2017). Owing to their profound influence on the regulatory processes that control disease progression, piRNAs are increasingly recognized as promising biomarker candidates (Zhang et al., 2021).

### Activin receptor signaling pathway

The activin receptor complex plays a role in decreasing neuroinflammation and stimulating oligodendrocyte progenitor differentiation (De Berdt et al., 2018). Specifically, *activin A* is a member of the *transforming growth factor β superfamily*, and it is crucial in cell proliferation, migration, differentiation, and immune regulation (Link et al., 2016; Li Y. et al., 2022). It is also synthesized in neurons, thus playing a role in neuroprotection. *Activin A* binds to type II receptors, which enables type I receptors to phosphorylate *Smad family member 2* (*SMAD2*) and *Smad family member 3* (*SMAD3*), which are intracellular signaling proteins. These signaling proteins form the SMAD (abbreviated from the *Caenorhabditis elegans Sma* genes and the *Drosophila Mad*, mothers against decapetaplegic genes) complex by assembling with *Smad family member 4* (*SMAD4*), and this complex regulates the expression of specific genes in the brain (Zheng et al., 2017).

In AD models, activin was found to induce neuronal development and neurite outgrowth in transgenic mice overexpressing familial AD mutations (Park et al., 2016). AD mice exhibited decreased neuronal development, but was recovered through the addition of activin A. *In vitro* models have also shown that *activin A* produced from nerve fibroblasts facilitate the migration and proliferation of Schwann cells, implicating its role in peripheral nerve repair (Li Y. et al., 2022). *Hsa-let-7 g-5p* is a circulating miRNA that has been found to be downregulated in AD (Kumar and Reddy, 2016; Kafshdooz et al., 2023). Neuronal loss in the brain has also been found in the downregulation of *let-7 g* (Derkow et al., 2018). However, it’s active role in the pathophysiology of AD is yet to be described.

### Actomyosin contractile ring organization

Neurons rely on the cytoskeletal networks for its morphology due to its highly polarized nature (Kang and Woo, 2019). The microtubule system and actin microfilaments in the cytoskeleton largely affect synapse morphology, which impacts synapse function (Torres Cleuren and Boonstra, 2012). Actin dynamics, including polymerization, depolymerization, severing, and bundling, modulate synaptic plasticity (Kang and Woo, 2019).

Tau proteins, which accumulate to form neurofibrillary tangles, accumulate in the axons in brain tissue by binding to microtubules. In AD patients, there is a lower affinity for microtubules and higher tau polymerization. In addition, the *actin-depolymerizing factors (ADF)/cofilin family* play a large role in regulating the actin dynamics in the brain (Torres Cleuren and Boonstra, 2012; Kang and Woo, 2019; Rahman et al., 2020; Bamburg et al., 2021). Cofilin functions to enhance actin polymerization, enhancing nucleation and filament formation.

In AD patients, ADF/cofilin-actin rod aggregates have been detected in amyloid plaques. These aggregates were determined to aid in tau recruitment, resulting in defects in axonal and vesicular transport (Torres Cleuren and Boonstra, 2012; Bamburg et al., 2021). *MiR-15b-5p* targets mRNA involved in cell cycle regulation and apoptotic pathways. It is downregulated in AD patients, compared to controls (Wu et al., 2020). In line with this, *in vitro* models showed that *miR-15b* inhibits the expression of *BACE1* and *APP* (Li and Wang, 2018; Liu et al., 2019).

### AGE-RAGE signaling pathway

AGEs are proteins or lipids that are glycated with sugar molecules. They have been linked with AD, such that patients exhibit increased levels of AGEs compared to healthy controls (Cai et al., 2016). AGEs interact with their multi-ligand cell receptors RAGE, which leads to the formation of reactive oxygen species (ROS). Due to the role of AGE-RAGE signaling in the formation of ROS, a link between AD and diabetes has been hypothesized. This association may be due to various factors, including insulin resistance, chronic inflammation, impaired glucose metabolism, and oxidative stress. The blood brain barrier is disrupted by the formation of ROS, which results in dysfunction and stimulates neuroinflammation that may progress to AD (Dhapola et al., 2024).

The glycation of proteins and sugars contribute to the development of age-related AD by triggering the AGE-RAGE axis, promoting synaptic dysfunction, and triggering oxidative stress response. An accumulation of AGEs, due to the imbalance in its generation and clearance, has been found to affect the permeability of the mitochondrial membrane, effectively resulting in oxidative stress (Dhapola et al., 2024).

An increased level of AGEs were also correlated with decreased cognitive and functional performance (Cai et al., 2016; Prasad, 2019). Imaging studies have found that AGEs localize in the senile plaques and extracellular spaces in AD patients (Khoury and Luster, 2008). The *let-7b-5p* miRNA mediates apoptosis caused by hypoxic spinal cord injury (Piscopo et al., 2018). It has been found to be downregulated in a study involving blood biomarkers in AD patients (Leidinger et al., 2013).

## Conclusion

This systematic review emphasizes the pivotal role of ncRNAs in the molecular pathology of AD, underscoring their potential as biomarkers and therapeutic targets. As the field of ncRNA research continues to develop, it holds the promise of unveiling novel strategies for the diagnosis, treatment, and management of AD, contributing to better outcomes for individuals affected by this devastating neurodegenerative disorder. The findings from this systematic review suggest that ncRNAs hold promise not only as biomarkers for early detection and diagnosis of AD but also as novel therapeutic targets. Although, our study is limited by several factors. The AD diagnosis criteria, AD type, and technology used varied across the studies included in the review. The ethnicity of the participants also varied, potentially affecting the generalizability of the results. Additionally, there was a noticeable difference in the number of studies involving miRNAs compared to lncRNAs, circRNAs, and piRNAs. As such, future studies can highlight the association of lncRNAs, circRNAs, and piRNAs with the risk of AD. More detailed studies on how ncRNAs are involved in AD are needed to identify the role of these potential biomarkers as predictive and therapeutic targets. Identifying the mechanism of action of ncRNAs is also crucial to identify how they can serve as treatment targets in the long run. As seen in this review, the identification of specific ncRNAs associated with AD pathogenesis, such as those involved in the Activin Receptor Signaling Pathway, Actomyosin Contractile Ring Organization, and AGE-RAGE Signaling Pathway, provides valuable insights into the molecular mechanisms driving the disease. These insights pave the way for the development of targeted interventions that could potentially modulate the activity of these ncRNAs, thereby offering new avenues for the treatment and management of AD. Furthermore, the systematic review highlights the importance of advancing our understanding of ncRNAs in AD through further research. It is imperative to validate the roles of these ncRNAs in larger and more diverse populations to strengthen their utility as clinical biomarkers and therapeutic agents. Additionally, exploring the intricate network of interactions among different types of ncRNAs and their combined impact on AD pathology could unveil new dimensions of the disease mechanism.

## Data availability statement

The original contributions presented in the study are included in the article/Supplementary material, further inquiries can be directed to the corresponding author.

## Author contributions

RC: Conceptualization, Data curation, Formal analysis, Funding acquisition, Investigation, Methodology, Project administration, Resources, Supervision, Validation, Writing – original draft, Writing – review & editing. JSy: Conceptualization, Data curation, Formal analysis, Investigation, Methodology, Project administration, Supervision, Validation, Writing – original draft, Writing – review & editing. CD: Methodology, Writing – review & editing. CC: Methodology, Writing – review & editing. LD: Methodology, Writing – review & editing. BP: Methodology, Writing – review & editing. WP: Methodology, Writing – review & editing. JY: Conceptualization, Methodology, Writing – review & editing. JV: Conceptualization, Methodology, Writing – review & editing. JSe: Conceptualization, Methodology, Writing – review & editing. AG: Funding acquisition, Writing – review & editing.

## Supplementary material

SUPPLEMENTARY FIGURE 1 Number of independent original published studies on non-coding RNAs (ncRNAs) with respect to Alzheimer’s disease (AD) from 2007 to 2023. From only two studies in 2007, the number of studies has increased to more than 25 from 2020 onwards, highlighting the increasing awareness and potential of ncRNAs as possible biomarkers or therapeutic targets for AD.

SUPPLEMENTARY FIGURE 2 Total number of cases and control participants of the included studies according to age. Whether case or control, most of the participants in the study population of the included studies in this systematic review were more than 65 years old.

SUPPLEMENTARY TABLE 1 Keywords and search items used for each database.

SUPPLEMENTARY TABLE 2 Compiled studies included in this systematic review.

SUPPLEMENTARY TABLE 3 Compiled unique ncRNAs that are associated with Alzheimer’s disease.

SUPPLEMENTARY TABLE 4 Pathway enrichment analysis of gene targets of the top miRNAs.
