# Supplementary figures and images for: Non-coding RNAs involved in the molecular pathology of Alzheimer’s disease: a systematic review
Source: Front Neurosci. 2024 Jun 28;18:1421675. doi: 10.3389/fnins.2024.1421675 (PMC11243705; doi:10.3389/fnins.2024.1421675)

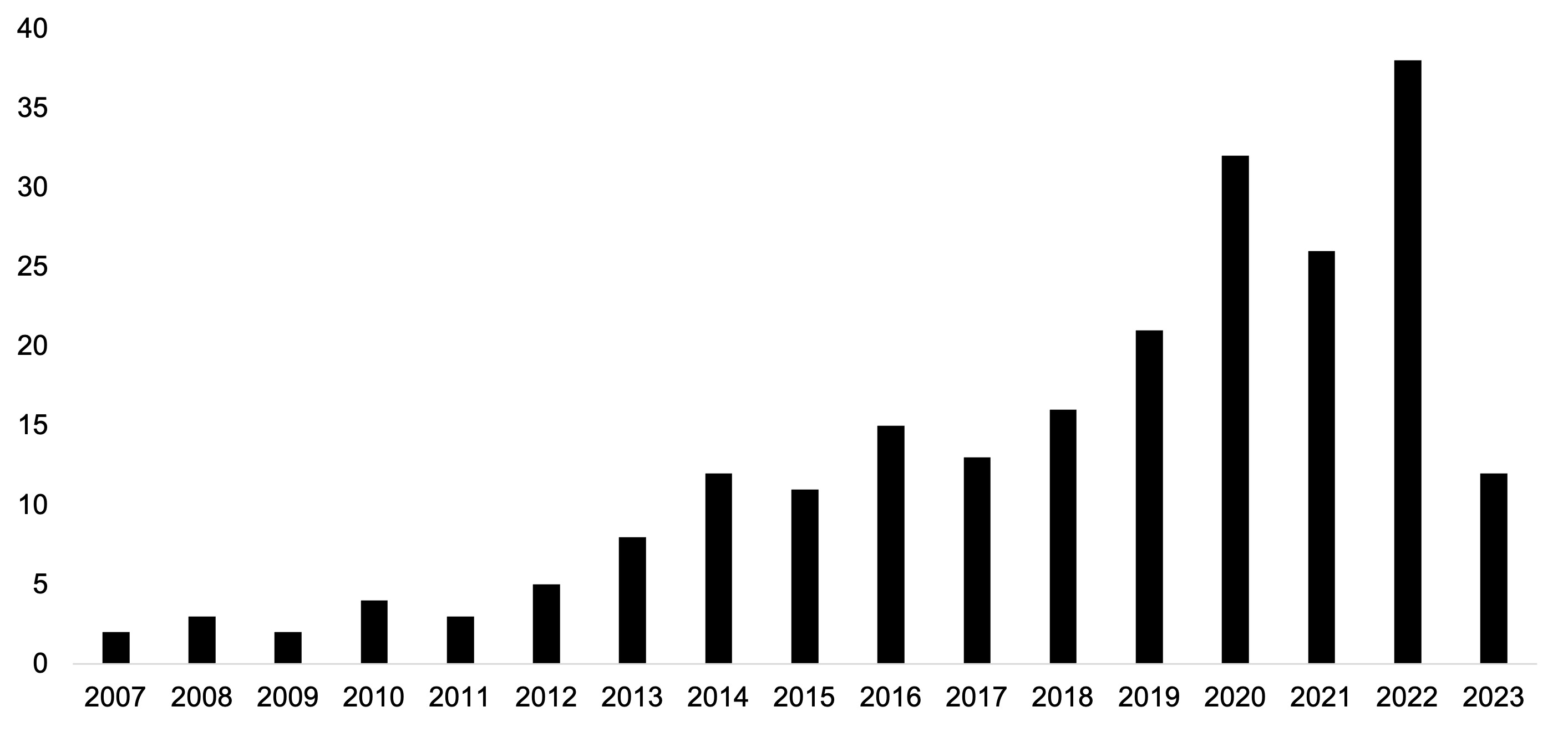

Supplement: Supplementary file 1 [file Data_Sheet_1.zip › Supplementary Material/Supplementary Material Presentation/Supplementary Figure 1.jpg]

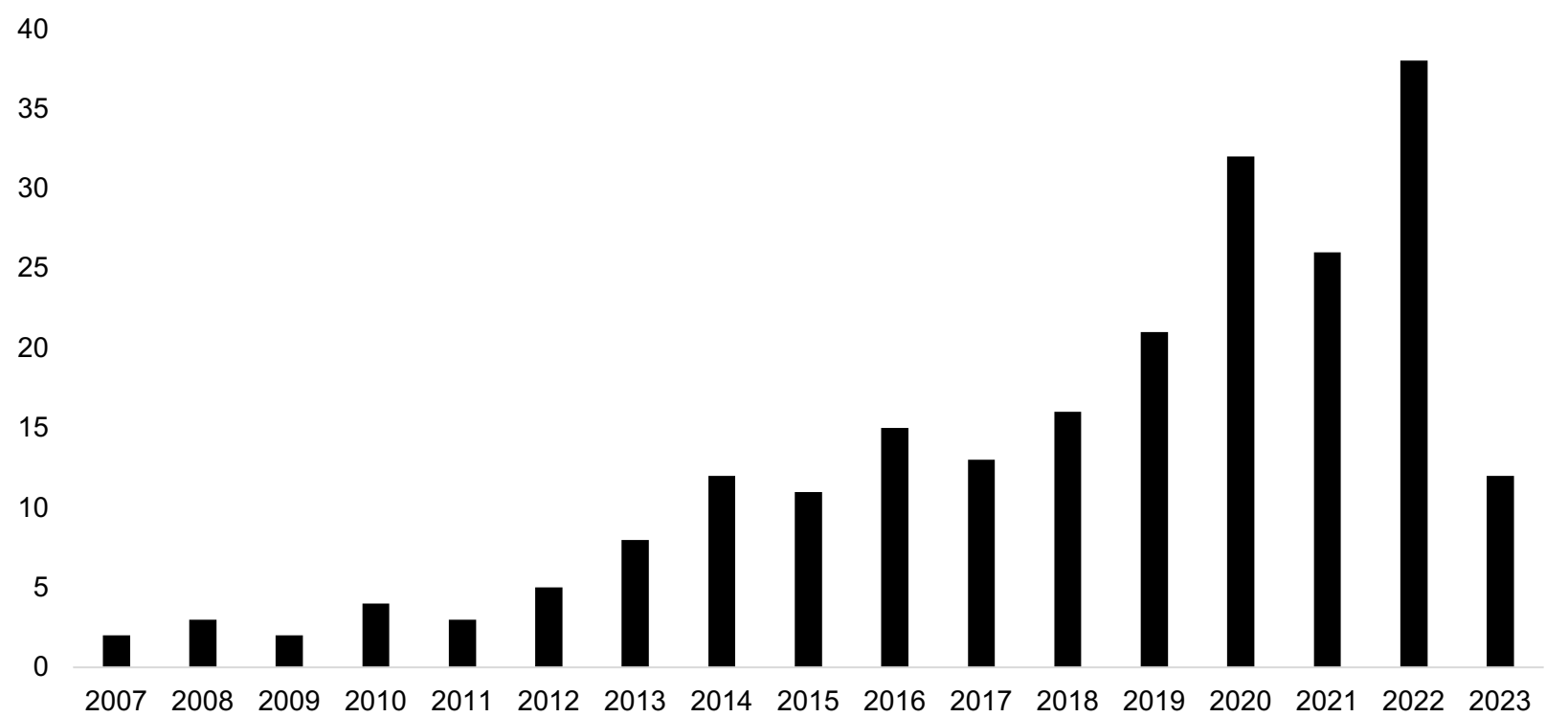

Supplement: Supplementary file 1 [file Data_Sheet_1.zip › Supplementary Material/Supplementary Material Presentation/Supplementary Figure 1.pdf]

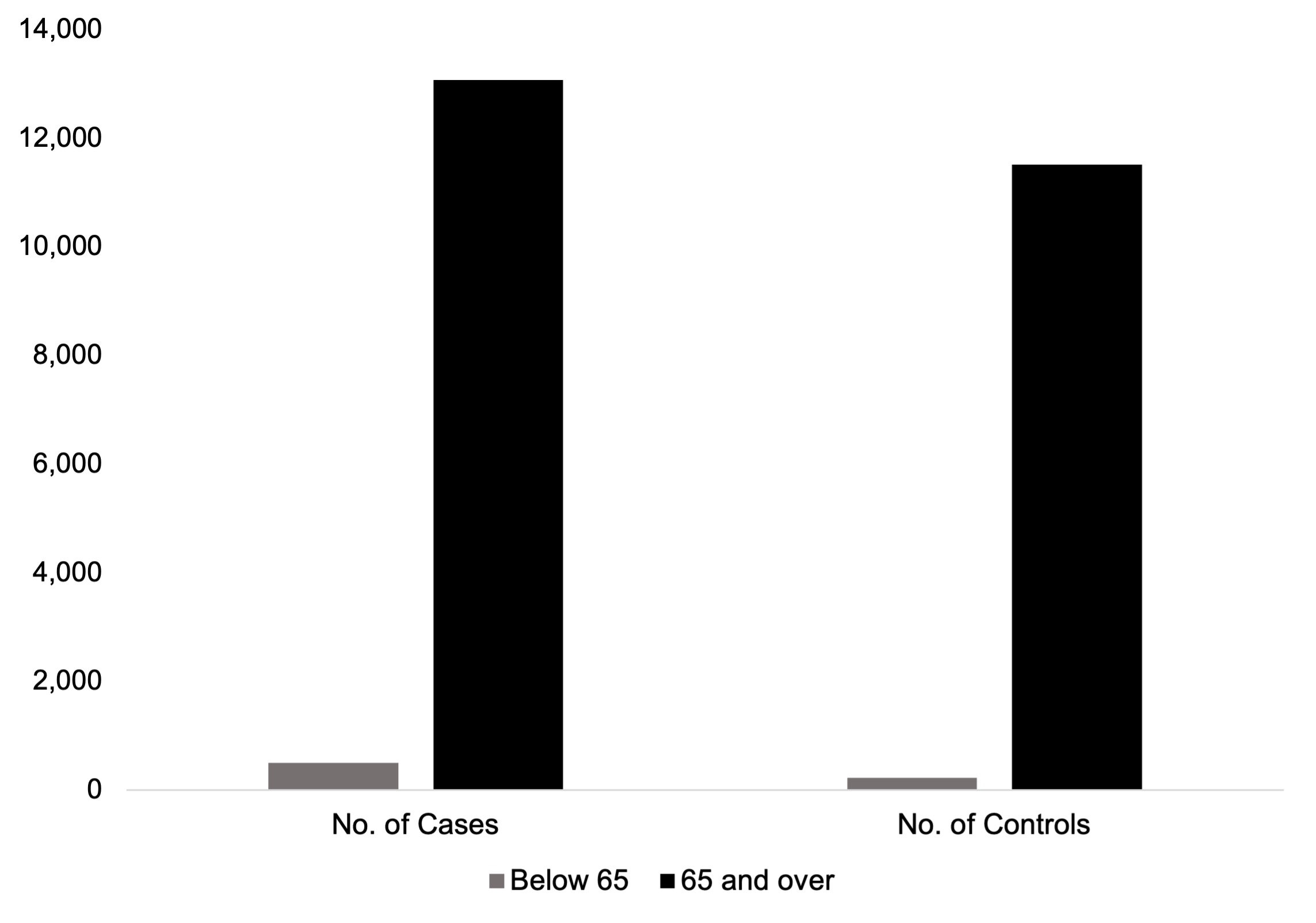

Supplement: Supplementary file 1 [file Data_Sheet_1.zip › Supplementary Material/Supplementary Material Presentation/Supplementary Figure 2.jpg]

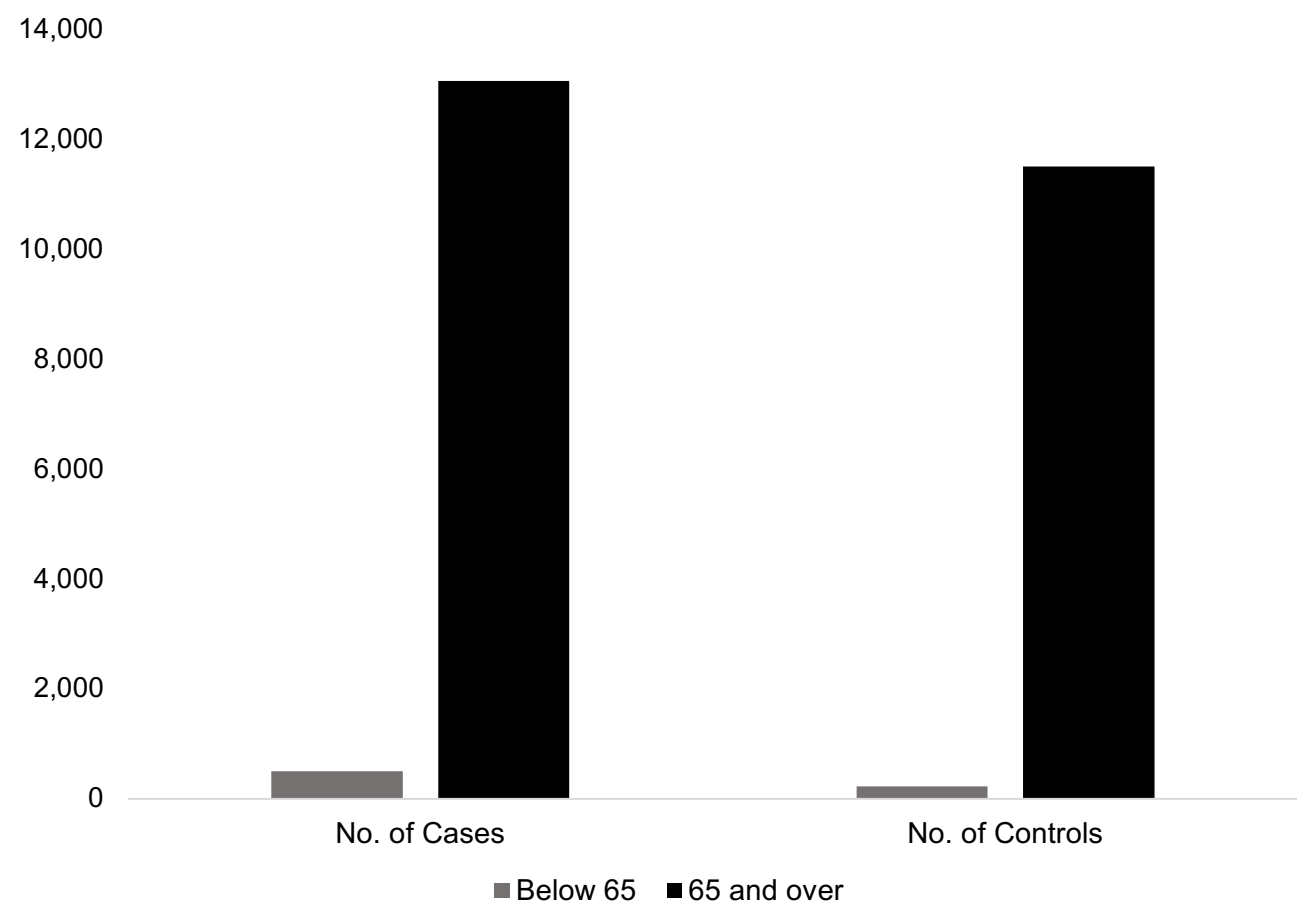

Supplement: Supplementary file 1 [file Data_Sheet_1.zip › Supplementary Material/Supplementary Material Presentation/Supplementary Figure 2.pdf]
